# The Dopamine Dancer: The Value of Holistic Autotherapy in Parkinson's Disease

**DOI:** 10.1002/mdc3.14166

**Published:** 2024-08-02

**Authors:** Anne Hellevik, Karin Wirdefeldt, A. J. Lees

**Affiliations:** ^1^ Citizen Scientist Stockholm Sweden; ^2^ Department of Clinical Neuroscience and Department of Medical Epidemiology and Biostatistics Karolinska Institutet Stockholm Sweden; ^3^ Department of Neurology The National Hospital, Queen Square London United Kingdom

**Keywords:** dance, meditation, remission, Parkinson's disease

In 2013 one of us (Andrew J. Lees) described 2 women who “recovered” from Parkinson's disease after several years of slowly worsening symptoms. Both were able to slowly discontinue levodopa (l‐DOPA) without relapse and remained asymptomatic for more than 12 months before being discharged from the clinic. One of them was found to have a reduced fluorodopa uptake, using positron emission tomography, at a level intermediate between that found in healthy controls and that seen in people with Parkinson's disease The clinical picture in both cases was incompatible with functional parkinsonism, and reversible secondary causes had been excluded. In 1 of them symptoms improved after the resolution of a period of sustained domestic mental stress, whereas in the other bradykinesia and rigidity appeared within 2 weeks of open heart surgery.^1^ The same author (Andrew J. Lees) had also diagnosed another patient with Parkinson's disease who before medical treatment was started lost all his signs and symptoms leading to a temporary retraction of the diagnosis. Facial hypomimia and bradykinesia and rigidity in the limbs then returned several months later and persisted. This patient remained under follow‐up for the next 12 years and responded well to l‐DOPA but developed motor fluctuations after 5 years of treatment. Although “remission” of symptoms in the early stages of disease followed by recurrence is not formally recorded in the literature, informal discussion with other experienced clinicians suggests that this is not uncommon and is not always explained by misdiagnosis (psychomotor retardation due to depression, iatrogenic or functional parkinsonism).

The subject of this case report, Anne Hellevik was diagnosed with Parkinson's disease in 2018, and through a structured program of dance, meditation, yoga, and cognitive autotherapy continued over several years she has managed to gradually discontinue her l‐DOPA therapy, and at the same time achieve a striking improvement in her functional capacity. Her personal narrative is included in the report (see Supplementary Material Data S1) along with a sequence of video clips, which demonstrate her remarkable degree of improvement.

## Medical Case History of Anne Hellevik

Anne Hellevik is a 60‐year‐old right‐handed Swedish woman who developed anxiety, with episodes of excessive perspiration and weight loss in 2014. A year later she became aware of stiffness in her left arm, neck, and shoulder. She then started to drag her left leg (see Video 1) and by 2016 had stress‐induced tremor at rest in the left arm and left leg. By 2018 she had begun to scuff the sole of her left foot on the ground, was taking shorter steps, and had developed a noticeable limp (see Video 2). Her left arm slowly became weaker and more shaky and involuntarily flexed at the elbow when walking, as if she were holding a handbag. She also noticed that sometimes the fingers of her left hand would involuntarily flex. The shaking in her left hand worsened; she had episodes of trembling of her jaw, leg, and body, and complained of distressing internal tremors especially at night. A painful restriction of her left shoulder made it difficult for her to lift her arm above her waist. She had double vision with a squint (diagnosed as intermittent esotropia), persistent excessive sweating, and nocturia (getting up 4 times each night to pass urine).

**Video 1:** Anne Hellevik in 2016, dog training 2 years before diagnosis showing reduced left‐arm swing, slowness in turning, reduced steppage gait, and a flexed posture.

**Video 2:** Anne Hellevik in 2019, dog training in an “off phase” a few months after starting levodopa showing a reduced steppage gait, rest tremor of left hand, marked flexion at the left elbow, and flexion of trunk and neck when walking.

**FIG. 1:**
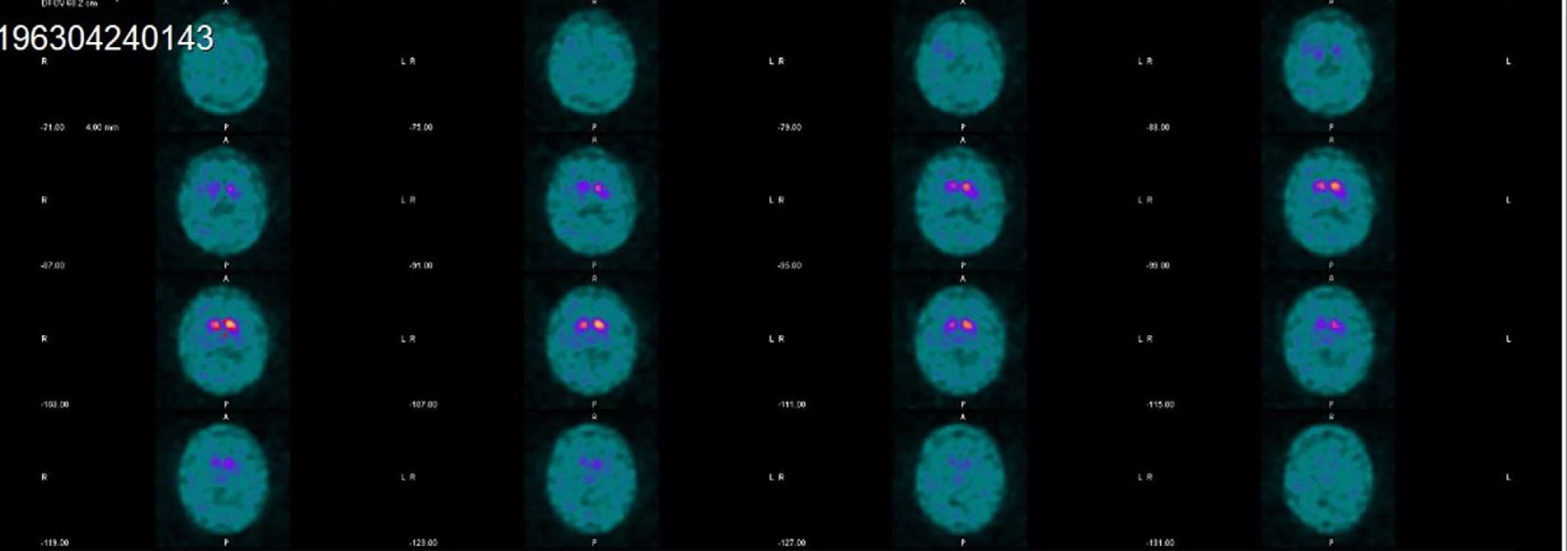
(DAT [dopamine transporter]) SPECT (single‐photon emission computed tomography) scan showing bilateral reduction in dopamine transporter isotope in the putamen more marked on the left and compatible with nigrostriatal dopamine denervation.

In 2018 she was diagnosed with Parkinson's disease by her general practitioner, which was later confirmed by 2 neurologists (including one of the authors Karin Wirdefeldt). In February 2019, she started Madopar (levodopa/benserazide), and within a week she felt more invigorated and noticed that the movements of her left side were quicker and that her stiffness had disappeared. She could also walk more freely and rapidly. Over the next few months the dose was increased slowly to Madopar (l‐DOPA/benserazide) 25/100, 4 times daily, and Madopar (l‐DOPA/benserazide) dispersible tablets 12.5/50 mg twice daily. By June 2019 she had already started to notice that each dose of Madopar provided good benefit but that its effect lasted only between 1 and 3 hours. During the “off period” troughs between doses, her mood was low and her tremor and stiffness were more severe than before treatment. There were no drug‐induced dyskinesias. Pramipexole MR was then introduced to help her tremor and increased to 1.31 mg per day. On examination she now had a mild left‐sided rest tremor of the hand and leg, mild left postural tremor of the hand, moderate bradykinesia in her left hand and foot on tapping and mild rigidity of the left arm, loss of left‐arm swing when walking, and milder right‐sided motor impairment (Karin Wirdefeldt). Investigations included a dopamine transporter scan, which revealed bilateral reduction in striatal uptake, slightly worse on the right, and greater in the putamen than the caudate nucleus, compatible with bilateral nigrostriatal dopamine denervation (see Fig. 1); a computed tomography (CT) head scan, an electroencephalogram, and sensory and motor nerve conduction studies and quantitative sensory threshold tests were all normal. She had a maternal uncle who had died with parkinsonism at the age of 77, 8 years after diagnosis.

Anne Hellevik then decided on her own initiative to begin reducing her medication, and by June 2020 she had stopped l‐DOPA completely. She told her neurologist and nurse specialist through remote consultations that occurred by necessity during the COVID pandemic that she was feeling a great deal better, had stopped limping, and was moving more easily. She next started to cut back on pramipexole, but even small dosage reductions led to increase in her tremor. By April 2021, apart from some intermittent morning left‐sided tremor, she was asymptomatic. The following year a Unified Parkinson's Disease Rating Scale (UPDRS), Part 3, score of 18 was recorded, and in 2023 the UPDRS, Part 1, score was 3; the UPDRS, Part 2, score was 3; and the UPDRS, Part 3, score was 23. In February 2024 she was reviewed by Karin Wirdefeldt and found to be strikingly improved compared to 4 years earlier, with several of her earlier symptoms now in remission (see Video 3). She was taking pramipexole MR 0.52 mg daily and biperiden 2 mg. On examination she had some left‐sided bradykinesia on finger tapping and foot stamping, tremor, and rigidity but had a normal gait, speech, and balance. Her Hoehn and Yahr score was stage 2, and her UPDRS, Part 3, score was 28, but it was noted that she was having a particularly bad day and most of the score was related to increased tremor (see Video 4). A further assessment in June 2024 the case notes mentioned that she formally exercises more than 420 minutes each week and does high‐intensity workouts, including dancing and skipping, for at least 1 hour a day (see Videos 5 and 6). Her UPDRS, Part 1, score was 1; UPDRS, Part 2, score was 0; and UPDRS, Part 3, score reduced to 24.

**Video 3:** Anne Hellevik in 2024, dog training showing only reduced left‐arm swing and greatly improved posture and speed of walking while taking only pramipexole MR 0.5 mg daily.

**Video 4:** Anne Hellevik in 2024, showing normal right‐finger taps and mild bradykinesia on the left, mild hypomimia, and normal speech and absent tremor (mirror view).

She has a normal sense of smell. There are no symptoms suggestive of rapid eye movement sleep behavior disorder although she had occasional nonfrightening visual hallucinations in 2022, which disappeared immediately after pramipexole dose reduction. She has no constipation, and there is no past medical history of depression. She is a nonsmoker who has abstained from alcohol since the diagnosis of Parkinson's disease. She used to work as a payroll administrator and is married with 2 children.

## Discussion

Although it is accepted that sustained striking symptom improvement can occur with l‐DOPA in Parkinson's disease, especially in young‐onset patients, a disappearance of all symptoms and signs within a few days of starting l‐DOPA would make a neurologist question the accuracy of the diagnosis. There are also a few patients who have a sustained long‐duration response to l‐DOPA without developing treatment complications, and who when examined have no bradykinesia, rigidity, or tremor. This may result in uncertainty about the accuracy of the diagnosis and require a cautious drug holiday, an apomorphine or l‐DOPA challenge test, and, if doubt remains about the diagnosis, a dopamine transporter single‐photon emission computed tomography (SPECT) scan to revalidate the diagnosis of Parkinson's disease.

Kinesia paradoxa was first reported as a response to life‐threatening events^2^ such as a shipwreck or fire, but spectacular temporary improvement in symptoms and signs can also be triggered by visual, emotional, and motivational cues.^3^, ^4^ A few patients also report experiencing whole days when all their symptoms disappear when they feel no need to take their regular antiparkinsonian medication. More commonly full mobility associated with a feeling of clear headedness can occur for up to an hour after waking, allowing patients to delay their first dose of medication for several hours.^5^

In 2005 the world press reported that Sister Marie Simone‐Pierre, a 44‐year‐old nun, had been cured of Parkinson's disease after members of her religious community had prayed for the intervention of Pope John Paul II, who himself had suffered for many years from Parkinson's disease. Her symptoms had started when she was 28 with mild tremors; in her thirties she had begun to notice stiffness in her legs, difficulty smiling, and problems in writing. She then noticed changes in her voice and experienced insomnia and pain. After the death of Pope John Paul II, her symptoms worsened, and she found it increasingly difficult to walk, making it likely she would have to stop her duties at the convent. After the prayers of some of her sisters and during a moment of eucharistic worship she explained that she had been overcome by a powerful force, which was followed by the rapid disappearance of all her symptoms. After a number of independent medical examinations and tests, including scans, her complete sustained recovery was confirmed by the Vatican as a miracle. She has now been free of symptoms without antiparkinsonian medication for 18 years.

The difficulty in accepting this account as a bona fide cure is that no details of her neurologist's findings or images or films taken while she had Parkinson's disease have been made available for public scrutiny, making it impossible to exclude a diagnosis of functional Parkinson's syndrome (https://www.theguardian.com/world/2007/mar/31/catholicism.france).

**Video 5:** Anne Hellevik in 2024, off dopaminergic medication dancing to Jackie Wilson's “Your Love Takes Me Higher.”

One of us (Andrew J. Lees) communicated with Sister Marie Simone‐Pierre by mail. She confirmed that before her sudden cure she had difficulty standing without support, could not drive, and had become very slow in all her movements. She concluded her letter as follows: “I do not wish to go any further. The doctors did their job and Rome recognised the miracle.”

In 2016 Smart and colleagues^6^ reported the case of a 78‐year‐old man who presented with a kinetic left‐hand tremor, shuffling gait, stooped posture, and falls. Despite an initial beneficial response to l‐DOPA, his symptoms worsened over the next 3 years, but after 12 years of follow‐up they remitted spontaneously. At the time of his diagnosis, a dopamine transporter scan was abnormal with reduced uptake in the right putamen and a CT head scan was normal. Between 2003 and 2012 he was seen annually by a neurologist and was maintained on a daily dose of 600 to 800 mg of l‐DOPA. His functional impairment remained stable, and on examination he had an intermittent bilateral rest tremor, mild bilateral bradykinesia, and cogwheel rigidity at the wrists. Some doubt about his responsiveness to medication was raised after several years of follow‐up, and after a formal l‐DOPA challenge test had revealed no benefit, it was decided to taper off his medication. This was achieved without any worsening of his symptoms. Two years after drug discontinuation the patient was asymptomatic and had only mild bradykinesia on finger taps and a mildly stooped posture on walking. Cogwheeling, rest tremor, and shuffling of gait noted on earlier neurological assessments had disappeared. As a young man the patient had been a postulant in a Franciscan monastery, where he had learned a silent form of meditative prayer, which involved focusing his mind on a single religious word to reach a meditative state. The patient reported to his doctors that he felt less “Parkinson's‐like” during meditation and that after his diagnosis he had started to reinvest energy in the practice.

**Video 6:** Anne Hellevik in 2024, off dopaminergic medication dancing to Donna Summer's “I Feel Love.”

Dance is a creative art form that brings pleasure to people all over the world. For people with Parkinson's disease, however, it has benefits that go far beyond socializing, creative expression, and physical exercise. A person with Parkinson's disease may enter a dance hall shuffling and stiff, but as soon as the music starts slowness and stiffness vanish. Dancing induces an intense feeling of exhilaration and freedom and restores large fast movement.^7^, ^8^ A recent systematic review reported that dance also appeared to be a highly promising treatment for depression, with large effects found compared with other forms of exercise particularly in young women.^9^

Anne Hellevik meditates in a dark room before bedtime, and on waking, lying down in bed with eyes closed for between 1 and 2 hours a day. She begins with heavy breathing to build up energy and then focuses on manipulating the flow of energy through the 7 chakras. She employs visualization to feel the space her body occupies in the universe and to elevate her emotions.^10^, ^11^ Through meditation she enters a new dimension, free from “her old self.” The state of mind resembles one she has also occasionally felt while being totally immersed in the natural world.

Despite the impressive improvement in symptoms achieved by Anne Hellevik with minimal medication, we are not advocating the abandonment of dopaminergic therapy in people who have responded well to treatment. We also remind physicians and patients that caution is necessary in the discontinuation of dopaminergic and anticholinergic medication even when it appears to the patient and physician that it is having no effect. Abrupt withdrawal of antiparkinsonian medication is known to sometimes lead to severe worsening and even death.

A marked improvement in symptoms lasting several years may seem to be incompatible with a neurodegenerative disorder. It is possible nonetheless that meditation, dance, and positive thinking may all energize the emotional locomotor system by activating the meso‐cortico‐limbic dopamine system and limbic cortex and so override the characteristic disturbance of movement in Parkinson's disease.^12^ This case of Anne Hellevik, the dopamine dancer, also illustrates the extent of improvement that can be achieved by self‐motivation, hopefulness, and positive thinking, and serves to remind physicians to be nuanced and circumspect when discussing the prognosis and course of Parkinson's disease at the time of diagnosis.

## Author Roles

(1) Research project: A. Conception, B. Organization, C. Execution; (2) Statistical analysis: A. Design, B. Execution, C. Review and critique; (3) Manuscript preparation: A. Writing of the first draft, B. Review and critique.

A.H.: 1A, 3B

K.W.: 1B, 1C, 3B

A.J.L.: 1A, 1B, 1C, 3A, 3B

## Disclosures

**Ethical Compliance Statement:** We confirm that we have read the journal's position on issues involved in ethical publication and affirm that this work is consistent with those guidelines. We confirm that the first author Anne Hellevik is the patient described in the manuscript and provided the videos, her personal narrative, and the details of her medical assessments.

**Funding Sources and Conflicts of Interest:** No specific funding was received for this work. The authors declare that there are no conflicts of interest relevant to this work.

**Financial Disclosures for the Previous 12 Months:** The authors declare that there are no additional disclosures to report.

## Supporting information


**Data S1.** The patient's narrative.
