# Supplementary material for: The Dopamine Dancer: The Value of Holistic Autotherapy in Parkinson's Disease
Source: Mov Disord Clin Pract. 2024 Aug 2;12(3):375–9. doi: 10.1002/mdc3.14166 (PMC11952934; doi:10.1002/mdc3.14166)
Supplement: Supplementary file 1 — Data S1. The patient's narrative. [file MDC3-12-375-s001.docx]

Supplementary Material

**The Patient’s Narrative**

I was diagnosed with Parkinson’s disease in 2019 but it is probable that my illness began five years before that (see video 1) After I received the diagnosis I was in a state of emotional shock and denial . Surfing the internet and reading about Parkinson’s made me feel much worse and increasingly hopeless. When I Googled ‘Can you get better from Parkinson’s disease’ the search engine responded with ‘Do you mean instead? ” You can’t get better from Parkinson’s? It was if I had been given a life-sentence.

I started on levodopa in 2019 and had an excellent response to the medication with a big improvement in stiffness, slowness and walking but within a few months I started to experience peaks and troughs. . Seven months after starting medication I decided I needed to regain control, and without notifying my doctors I started to tail off my medicine.

I gave myself 2 years to see if I could modify the course of my illness using as little medication as possible. I read uplifting stories from other people with Parkinson’s disease and took careful note of what each person had found helpful in reducing their symptoms. I was greatly influenced by Dr Joe Dispenza, author and citizen scientist, particular his meditation techniques that involved ‘blessing of the chakra energy centres’.(2, 3) I also began working on getting rid of past mental traumas and acquired techniques to expel all negative thoughts. I meditated for 1-2 hours every day and after about six months began to experience feelings of well-being and positivity that carried on for most of the day. I also did yoga and a challenging physical training programme that involved visualisation techniques to improve my gait, posture, dexterity, balance and stiffness. The most effective and rewarding form of exercise has been dance. Sometimes I will hear a tune by chance on the computer or radio which inspires me to dance spontaneously. I find music with a strong regular beat and fast rhythm especially motivating and energising ( e.g. Lady Gaga, Northern Soul and Techno). Sometimes I dress up before dancing and plan the choreography in advance. When I start to move to the groove I feel happy and joyful, my self-esteem increases and my aches and pains vanish (see videos 5 and 6 ). The longer I dance the freer my movements become. There is also a carry-over feeling of well-being after I have stopped that can last most of the day. I try to get eight hours sleep every night and set myself new challenges as well as continuing my hobbies of dog training and making jewellery. I had stopped levodopa completely by May 2020 and after that I managed to cut back my pramipexole dose to 0.5mg once a day.

I can control internal stress much better now and have lost my fear. In the last two years there have been days, sometimes weeks, when I feel cured and am entirely free of all symptoms. At these times I jump out of bed without stiffness or tremor and have boundless energy. Friends and colleagues no longer consider me to be ill and comment spontaneously on my improved physical appearance. The shake of my left hand is aggravated by strong emotion but compared to five years ago it is less and the internal tremors have disappeared. Even on mornings when I wake with tremor I am able to thread a very thin needle and have no difficulty lifting objects or doing housework. It is now very unusual for me to shake in the afternoon or evening. I can type quickly with very few errors and write without difficulty. Overall I have improved over 90 per cent in my ability to carry out everyday tasks quickly and smoothly and am much better now than I was in 2018.

I am planning to continue this programme of meditation, dance and exercise. Faith has played a large part in my improvement and I needed great determination and patience to get to where I am now. When I become aware of the return of a lost skill I feel pleased and grateful. The fact that I have done something that I was told was impossible gives me extra satisfaction. I have also successfully been able to help another young woman with Parkinson’s disease through mentoring and teaching her the methods I have used.
